# Adiponectin in Chronic Kidney Disease

**DOI:** 10.3390/ijms21249375

**Published:** 2020-12-09

**Authors:** Jarosław Przybyciński, Violetta Dziedziejko, Kamila Puchałowicz, Leszek Domański, Andrzej Pawlik

**Affiliations:** 1 Department of Nephrology, Transplantology and Internal Medicine, Pomeranian Medical University in Szczecin, Powstańców Wlkp. 72, 70-111 Szczecin, Poland; jarpe85@gmail.com (J.P.); domanle@pum.edu.pl (L.D.); 2 Department of Biochemistry and Medical Chemistry, Pomeranian Medical University in Szczecin, Powstańców Wlkp. 72, 70-111 Szczecin, Poland; viola@pum.edu.pl (V.D.); kamila.puchalowicz@pum.edu.pl (K.P.); 3 Department of Physiology, Pomeranian Medical University in Szczecin, Powstańców Wlkp. 72, 70-111 Szczecin, Poland

**Keywords:** adiponectin receptors, atherosclerosis, bone, diabetes, dialysis, kidney, obesity, transplantation, vasculature

## Abstract

Adiponectin is the adipokine associated with insulin sensitization, reducing liver gluconeogenesis, and increasing fatty acid oxidation and glucose uptake. Adiponectin is present in the kidneys, mainly in the arterial endothelium and smooth muscle cells, as well as in the capillary endothelium, and might be considered as a marker of many negative factors in chronic kidney disease. The last few years have brought a rising body of evidence that adiponectin is a multipotential protein with anti-inflammatory, metabolic, anti-atherogenic, and reactive oxygen species (ROS) protective actions. Similarly, adiponectin has shown many positive and direct actions in kidney diseases, and among many kidney cells. Data from large cross-sectional and cohort studies showed a positive correlation between serum adiponectin and mortality in chronic kidney disease. This suggests a complex interaction between local adiponectin action, comorbidities, and uremic milieu. In this review we discuss the role of adiponectin in chronic kidney disease.

## 1. Introduction

Since its discovery, adiponectin has been identified as one of the key regulators involved in glucose and lipid metabolism. Further analyses have shown its anti-inflammatory and anti-apoptotic roles in human cells. It is produced predominantly in adipocytes. Human adiponectin protein has 244 amino acids (30 kDa), as well as the complex primary structure of a signal peptide, a hyper-variable region, a collagenous domain of 22 G-X-Y repeats, and a globular domain. It is present in human serum in relatively high concentrations, in three different structural forms: trimer (low molecular weight, LMW), hexamer (middle molecular weight, MMW), and 12–18-mer (high molecular weight, HMW). Another circulating, active form is globular adiponectin. It is generated by proteolytic cleavage of full-length adiponectin and has biological activity in humans [1]. Adiponectin concentrations are gender specific, with higher levels in females [2,3]. There are three known receptors for adiponectin: adiponectin receptor 1 (AdipoR1), adiponectin receptor 2 (AdipoR2), and T-cadherin. The first two receptors have a similar structure, with seven transmembrane domains, and an intracellular zinc binding motif capable of downstream signaling in the cell [3]. Novel research has also shown distinct ceramidase activity for the intracellular part of these receptors [4]. AdipoR1 is widely present in human cells, with the greatest numbers in skeletal muscle, AdipoR2 is mainly present in the human liver. Globular adiponectin has the highest affinity to AdipoR1, therefore in animal studies it acts mainly in muscle cells [1]. T-cadherin, lacking a transmembrane structure, is considered a binding protein for the high molecular weight (HMW) form. Some studies have indicated that T-cadherin is a major binding partner for adiponectin, and causes its accumulation in the heart, vascular endothelium, and skeletal muscle. The direct downstream effect of this binding is still under investigation [3,5,6]. Adiponectin may also bind to calreticulin, which is present on the surface of macrophages, and possibly other cells [7,8]. There are several factors regulating the expression of adiponectin gene (*AdipoQ*), such as: forkhead box O1 (FOXO1), peroxisome proliferator-activated receptor γ (PPAR-γ), CCAAAT-enhancer binding protein α, and sterol regulatory element binding protein 1c (SREBP-1c) [9]. Despite more than 20 years of constant studies on adiponectin function in animals and humans, an increasing quantity of data has been added recently. As an example, adiponectin might be one of the key mediators responsible for regular physical activity benefits in humans. Adiponectin connects energy balance regulation in the central nervous system and peripheral tissues [10]. Interestingly, animal studies have shown that adiponectin ameliorates the proinflammatory effect of saturated free fatty acid rich diet in hypothalamus. This is achieved mainly by suppression of microglia cell activation [11]. Other authors concluded that decreased serum adiponectin to leptin ratio is an indicator of adipose tissue inflammation and dysfunction and might predict cardiovascular risk in humans [12]. Adipocyte line studies have shown a direct HMW–adiponectin effect in reducing the local inflammation caused by glucolipotoxicity, via the APPL 1-AMPK pathway [13]. Adiponectin injection might also attenuate renal cell apoptosis in rats exposed to chronic intermittent hypoxia. In this study adiponectin reduced reactive oxygen species (ROS) generation and endoplasmic reticulum stress in the renal tissue [14]. It is a well-known fact that patients with chronic inflammatory states, such as rheumatoid arthritis or inflammatory bowel disease, have high serum adiponectin concentrations. This might be partly explained by a compensatory adiponectin expression, however animal and in vitro studies have shown that adiponectin might directly stimulate proinflammatory factor secretion in various cells, for example fibroblast-like synoviocytes, or neutrophiles of colonic lamina propria T-lymphocytes. Further studies differentiated the potential effect of different molecular forms of adiponectin in inflammation. Data suggest that, particularly HMW adiponectin and its globular form, contrary to low molecular weight (LMW) adiponectin, might also promote inflammation via dose-dependent NF-κB stimulation [15]. In the face of multipotential and complex adiponectin function, in this review we will be analyzing its role in chronic kidney disease and its complications.

## 2. Adiponectin Function

Adiponectin has traditionally been associated with insulin sensitization, reducing liver gluconeogenesis, and increasing fatty acid oxidation and glucose uptake. In the liver, it inhibits phosphoenolpyruvate carboxykinase and glucose-6-phosphatase. In skeletal muscle, it promotes beta oxidation and lowers lipid accumulation via activation of 5′-AMP-activated protein kinase (AMPK). This action is mediated by adaptor protein, phosphotyrosine interacting with the PH domain, and leucine zipper 1 (APPL1). Another key metabolic pathway of adiponectin function is peroxisome proliferator-activated receptor α (PPAR-α) activation, which also increases fatty acid oxidation in both muscle and liver and increases glucose uptake in the latter organ. Generally, AdipoR1 activation triggers AMPK, while AdipoR2 activation triggers PPAR-α. Globular adiponectin has its unique effects on muscle metabolism and proliferation. Animal and cell line studies have shown an important adiponectin role in muscle differentiation and regeneration, and also as an autocrine/paracrine factor [10,16,17]. Additionally, adiponectin exerts an important protective role against ROS, by increasing oxidative stress detoxifying enzymes in mice skeletal muscles, mainly through the peroxisome proliferator-activated receptor γ coactivator-1α (PGC-1α) pathway in mitochondria [18,19]. It has been shown that nonalcoholic fatty liver disease, chronic alcoholic fatty liver diseases, and chronic hepatitis C were associated with reduced serum adiponectin levels, decreased hepatic adiponectin receptor expression, and impaired hepatic adiponectin signaling [20].

Adiponectin also has a prominent anti-inflammatory function. It inhibits nuclear factor κB (NF-κB) signaling, tumor necrosis factor (TNF-α) secretion, and expression of adhesion molecules. Furthermore, it increases interleukin 10 (IL-10) and interleukin 1 receptor 4 (IL-1R4) production and promotes macrophage polarization into the anti-inflammatory M2 phenotype. Research on mice overexpressing adiponectin indicated a lower expression of other proinflammatory cytokines, including interleukin 12 (IL-12), interleukin 17B (IL-17B), and interleukin 21 (IL-21) in adipocytes and stromal vascular cells. There was also downregulation of cytokine expression, including intercellular adhesion molecule 1 (ICAM-1), C-C motif chemokine ligand 5 (CCL5/RANTES), granulocyte colony-stimulating factor (GCSF), granulocyte–macrophage colony-stimulating factor (GM-CSF), vascular endothelial growth factor receptor-1 (VEGFR-1), and thrombopoietin (TPO) in those cells [16,21]. Studies have also shown its anti-apoptotic role among various cells. Adiponectin attenuated oxidative stress caused by diabetes in cultured human umbilical vein endothelial cells through the cAMP/protein kinase A (PKA) pathway [22]. On the other hand, oxidative stress might block adiponectin secretion in murine adipocytes through PPAR-γ inhibition [9]. Another important aspect is the ceramidase activity of both AdipoR1 and AdipoR2 [3,23]. Analysis of these two receptors showed that, despite their difference in crystal structure, they are both capable of breaking down ceramide to sphingosine and free fatty acids [4]. After phosphorylation, sphingosine was turned into sphingosine 1-phosphate (S1P), an intracellular signaling molecule playing an independent part in insulin sensitization and metabolism in animal studies [24]. Serum adiponectin levels are significantly lower in many diseases, including lipodystrophy, type 2 diabetes (T2D), obesity, metabolic syndrome, and atherosclerosis. Higher than normal concentrations are observed in type 1 diabetes (T1D), and in diabetic nephropathy [9]. Adiponectin’s antiatherogenic properties have been proven in many studies. It inhibits smooth muscle cell proliferation, and decreases the expression of endothelial adhesion molecules, thus mitigating regional inflammation [25]. Adiponectin takes part in energy balance regulation, acting directly in the central nervous system. Animal studies have shown that hexameric and trimeric adiponectin forms can cross the brain–blood barrier, and that adiponectin receptors, AdipoR1 and AdipoR2, are present in the hypothalamus. Adiponectin activates AMPK in the hypothalamus and affects local proopiomelanocortin and neuropeptide Y signaling. Previous animal studies of adiponectin’s role brought conflicting results, showing anorexigenic or orexigenic effects [26,27]. Novel studies [28] have suggested that adiponectin action might depend on central nervous system glucose level. With high glucose concentration, adiponectin increased food intake through inhibition of proopiomelanocortin signaling. An opposite effect occurred with low glucose. Recent animal studies [29,30] have also shown adiponectin’s ability to relax gastric muscles, causing gastric distention, which might lead to early satiety and decrease food intake.

## 3. Adiponectin Action in Kidneys 

Adiponectin is primarily excreted by the liver and is assumed to play only a secondary role in the physiology of kidney clearance [31,32]. Due to the large molecular weight of the adiponectin monomer (28 kDa), only monomers and dimers may cross the glomerular filtration barrier and be found in urine. Adiponectin is present in the kidneys mainly in the arterial endothelium and smooth muscle cells, as well as in the capillary endothelium, and to a lesser extent in epithelial cells in the brush border of proximal and distal tubules. Proximal tubular cells may also produce adiponectin, mainly in inflammatory conditions [31,33]. Adiponectin receptors can be found inside glomeruli: on endothelial cells, podocytes, mesangial cells, and Bowman’s capsule epithelium. They are also present in proximal tubular cells. Most of these receptors are AdipoR1 [34]. AdipoR1 mRNA is present in a 20 times higher concentration than AdipoR2 in human proximal tubular cells [35]. Komura et al. [36] performed a series of experiments on nephrectomized mice and reported impaired adiponectin clearance as the reason for higher adiponectin levels in kidney failure. Adiponectin secretion from adipose tissue did not change notably. No significant difference between the isoforms was found. Injection of nephrectomized mice plasma into mice with normal kidney function also impaired adiponectin clearance. Cystatin C has been identified as one of the toxins suppressing adiponectin function. Yu et al. [37] noted a significant increase of both serum and urea adiponectin and its receptors in the kidney tissue of rats 2 and 4 weeks after induced kidney injury. D’Apolito et al. [38] also observed an adiponectin increase in uremic mice. This effect was linked to an increase in ROS generation and was reversed by adding the artificial ROS scavenging enzyme MnTBAP. Adiponectin exerts a wide array of effects in kidney cells. Adiponectin increases the activity of AMP-activated protein kinase, which reduces podocyte permeability to albumin. This effect is caused by inhibition of NADPH oxidase Nox4 in podocytes and reduction of oxidative stress [39]. Similar effects have been found in tubular epithelial cells. AdipoR1 activation in this cell reduces ROS generation through NOX inhibition by two independent pathways: AMPK, and exchange protein activated by cAMP (Epac). This action might protect cells from the ROS injury induced by angiotensin II [40]. It has also been proven that antihypertensive treatment with angiotensin-converting-enzyme inhibitors (ACEI) or angiotensin II receptor blockers (ARBs) increases serum adiponectin levels in an unclear mechanism [41]. Similarly, globular adiponectin mitigates the inflammation in proximal tubular renal epithelial cell lines caused by uric acid. Yang et al. [42] demonstrated that this effect was conducted by AdipoR1 activation and AMPK signaling. Another major pathway affected by adiponectin is mechanistic target of rapamycin kinase (mTOR) signaling. Incubation with adiponectin decreased mTOR and ribosomal protein S6 kinase (S6K) phosphorylation in mesangial cell cultures exposed to high glucose concentrations, mimicking diabetes. In addition, in this model adiponectin partially blocked Smad2 and Smad3 phosphorylation in response to transforming growth factor β (TGFβ) stimulation, therefore attenuating tissue fibrosis. Furthermore, adiponectin mitigated NFκB signaling and oxidative stress in the same mesangial cells, and lowered monocyte chemoattractant protein-1 (MCP-1) density and macrophage infiltration in a diabetic *AdipoQ*^−/−^ mouse model [43]. Novel research has shown that adiponectin, probably through S1P generation, might mitigate glomerular inflammation by inhibition of NLR family pyrin domain containing 3 (NLRP3) inflammasome activation in podocytes [44]. Mice models have shown adiponectin accumulation in remnant kidney tissue after 5/6 nephrectomy. Even though this procedure did not significantly affect blood pressure or creatinine clearance, it caused glomerular hypertrophy and interstitial fibrosis. Interestingly, when researchers compared the effect of this procedure in adiponectin knockout and wild type mice, they found milder albuminuria and histological damage in the remnant kidney tissue of wild type animals. One of the mechanisms involved in this process is significantly lower nephrin expression in adiponectin knockout mice. Another negative process occurring more extensively in KO mice was macrophage infiltration due to overexpression of adhesion molecules, TNFα, collagen I/III, and TGFβ. KO mice also had a lower expression of NOX. Furthermore, adiponectin replenishment minimized the negative effects in adiponectin-KO mice after 4 weeks of observation, both in histological results and proteinuria [45]. Yuan et al. [46] injected an adiponectin vector into the peritoneum of Wistar rats. They did not find any differences in kidney function or TGFβ expression in healthy rats, with and without plasmid injection. The situation changed in rats with induced diabetes. Diabetic rats with adiponectin hyperexpression had lower proteinuria and less enlarged kidneys, with both decreased mesangial expansion and intraglomerular matrix deposits. There was also lower ROS generation in the plasmid injection group, mainly due to endothelial nitric oxide synthase (eNOS) overexpression in kidney tissue. In this model of early diabetic nephropathy, the diabetic rats had a serum creatinine similar to the control rats. Similarly, positive results of adiponectin overexpression on proteinuria, inflammation, and oxidative stress have been found in rats with induced diabetes [47]. Other authors developed a mice model of podocyte apoptosis. The apoptotic effect was generated after injection of a specific agent that caused dimerization of a mutated intracellular protein, which led to apoptosis via caspase-8 activation. The apoptotic effect was dose-dependent, giving the opportunity to simulate different clinical situations. Secondarily, mice were crossed to obtain adiponectin knockout and adiponectin overexpressing crosses. The results showed that adiponectin overexpression, in contrast to lack of adiponectin, promotes podocyte recovery after induced apoptosis. There was also decreased renal fibrosis in the adiponectin overexpressing group after 60 days of observation [48].

## 4. Adiponectin in Chronic Kidney Disease 

In the last two decades, many trials have shown the emerging role of adiponectin in kidney disease. The main cause of morbidity in this population remains cardiovascular disease (CVD). Other complications include malnutrition, atherosclerosis, chronic inflammation, and elevated oxidative stress [49,50,51,52]. Despite a negative metabolic status, patients with end-stage renal disease (ESRD) have two to three times higher serum adiponectin levels than subjects with normal kidney function. This is partly due to increased adiponectin secretion from adipose tissue. Serum adiponectin in patients on either peritoneal dialysis or hemodialysis is approximately three-fold higher than in the general population, and none of those methods remove adiponectin significantly [53,54]. Some studies pointed out that factors contributing to lower adiponectin secretion are oxidative stress and sympathetic nervous activity, which are common in chronic kidney disease [53]. There is also a negative correlation between visceral fat, and adiponectin production and plasma concentration. Nagakawa et al. [55] also found a negative association between the number of metabolic syndrome components and plasma adiponectin. Marinez Cantarin et al. [56,57] demonstrated increased AdipoR1 expression in the skeletal muscles of uremic patients, and elevated AdipoR1 mRNA expression in their adipose tissue. Furthermore, there was increased AMPK phosphorylation in skeletal muscles, but its secondary downstream effects were impaired. Both acetyl-CoA carboxylase phosphorylation and carnitine palmitoyl transferase-1 concentrations were decreased in skeletal muscles, causing reduced fatty acid oxidation. Similar negative effects have been observed after incubation of human myocyte cultures in uremic plasma. In contrast, Sopić et al. [58] suggested a downregulation of AdipoR1 in peripheral blood mononuclear cells in hemodialysis patients. Although these results are partially conflicting, they all suggest a possible blocking of adiponectin action in chronic kidney disease (CKD) patients. There is also a significant growth of serum adiponectin levels in diabetic nephropathy, particularly in patients with A3 albuminuria and advanced stages of diabetic nephropathy. This chiefly contributes to elevation of the HMW form of adiponectin, whose concentration is inversely associated with estimated glomerular filtration rate (eGFR). Higher levels of adiponectin are an independent risk factor for severity of diabetic nephropathy. Animal models have shown a decrease of AdipoR density and function in the kidneys of diabetic rats, but the results were inconsistent, especially in the case of AdipoR2, and need further elucidation [9]. 

## 5. Adiponectin Paradoxical Association with Mortality 

Whereas numerous studies of animal models and cell lines have shown the beneficial effects of adiponectin, controversially, those results do not match the majority of observational human studies. A retrospective analysis of the modification of diet in renal disease (MDRD) study showed that elevated serum adiponectin concentration was associated with an increased death risk. The study’s population featured 850 patients with stage 3 and 4 CKD, chiefly non-diabetic. A 10-year follow-up demonstrated a 3% increase in general mortality, and a 6% increase in cardiovascular mortality, with each 1 µg/mL increase of adiponectin concentration from the study’s baseline. Patients with higher adiponectin levels had lower body mass index (BMI), eGFR, and albumin, and higher blood pressure and proteinuria. Moreover, they were predominantly female patients. One of the explanations for the higher mortality associated with higher adiponectin concentrations might be an inverse relationship between BMI and mortality in patients with CKD [59]. The Hemodialysis (HEMO) study, which enrolled 182 hemodialysis patients, showed a non-linear association between adiponectin levels, and CVD occurrence or all-cause mortality. The highest risk for these events was found in the highest and lowest adiponectin concentration quadrants. The serum adiponectin level was inversely related with serum CRP levels and BMI, possibly as a compensatory mechanism. Lower adiponectin was also linked with pre-existing vascular disease at the baseline [60]. The prospective study of 501 hemodialysis patients (malnutrition, diet, and racial disparities in chronic kidney disease (MADRAD) cohort) also showed an unfavorable prognosis in the group with highest serum adiponectin concentration. In that study, adiponectin was related with serum high-density lipoprotein (HDL) cholesterol, and inversely related with body fat, lean body mass, triglycerides, and total and low-density lipoprotein (LDL) cholesterol. The authors calculated that a 10 µg/mL increase in serum adiponectin led to an approximately 25% increase in mortality risk [61]. In contrast, another study has shown an inverse relationship between CVD incidents and CVD mortality, and serum adiponectin among hemodialysis patients with end stage renal disease. This study showed a direct correlation between adiponectin and protective factors for CVD, such as HDL cholesterol and insulin sensitivity. On the other hand, the authors did not take into consideration the nutritional status of patients [62]. There are many other studies that have suggested better prognosis and lower incidence of CVD events in patients with higher plasma adiponectin concentrations and CKD at various stages [53]. However, Ortega Moreno et al. [63] observed an increased all-cause mortality rate in unselected patients with T2D and higher serum adiponectin levels. In this study, the positive relationship of adiponectin and mortality was only true in patients with eGFR ≥ 60 mL/min/1.73 m^2^. In patients with eGFR < 60 mL/min/1.73 m^2^, there was a strong confounding effect linked with low eGFR. A uremic milieu might also change adiponectin signaling and isoform proportions, causing target tissue adiponectin resistance. In protein–energy wasting (PEW) patients, higher adiponectin levels might not show beneficial effects, and be an indicator of negative prognosis. Some authors, depending on the animal model, have shown that adiponectin might have an anorexic effect in the central nervous system [64,65]. The possible direct negative effect of high serum adiponectin in these patients needs to be investigated. Indeed, Ortega Moreno et al. [66] facilitated a Mendelian randomization approach in a group of 356 patients with T2D and coronary artery disease. They observed a positive association between serum adiponectin and cardiovascular mortality. Moreover, allele A of the rs822354 adiponectin gene, a single nucleotide polymorphism linked with higher serum adiponectin, was an independent cardiovascular death risk factor. This suggests that increased adiponectin might have a causative, negative effect on CVD. A few studies have shown that, in some clinical scenarios (such as colitis or arthritis), adiponectin might in fact exacerbate inflammation [67]. Moreover, Wannamethee and colleagues [68] showed that elevated serum adiponectin is strongly associated with increased *N*-terminal prohormone of brain natriuretic peptide (NT-proBNP), an indicator of heart failure, where natriuretic peptides and fluid overload might increase adiponectin expression. Furthermore, Kim et al. [69] found that serum adiponectin is independently and inversely related to hemoglobin levels in patients with CKD, possibly impairing hematopoiesis.

## 6. Adiponectin and Nutritional Status 

There is growing evidence that the action of adiponectin in ESRD is significantly altered, and dependent on nutritional status. Choi and colleagues [70] found a positive correlation between baseline serum adiponectin and mortality in a cohort of elderly Asian patients. Interestingly, high adiponectin levels showed a strong synergistic effect with low BMI upon all-cause and cardiovascular mortality (hazard ratio of 6.25 and 13.89, respectively). There was also an inverse correlation between serum adiponectin and skeletal mass. Moreover, HMW adiponectin concentration remained strongly linked with mortality. One of the biggest challenges facing modern nephrology is obesity-related chronic kidney disease. Typical features of this disease are glomerulomegaly, proteinuria, focal segmental glomerulosclerosis (FSGS), and eventually kidney failure. Serum adiponectin levels are lower in obese patients, compared to those in lean patients, and are often accompanied by insulin resistance. Studies have shown that both lower adiponectin gene expression and decreased adipocyte secretion might explain this process. One of the factors linking kidney injury and obesity might be the AMPK inhibition caused by constant and excessive calorie intake. Physiologically, AMPK activation helps organisms to thrive during periods of fasting. This might be part of the crosstalk between the liver, kidney, and adipose tissue, which is vital for homeostasis, and partly mediated by adiponectin and fetuin-A (a protein produced in the liver) [71]. Experimental data have shown that kidney inflammation and fibrosis in mice on a high fat diet is associated with decreased AMPK function in kidney cells and might be mitigated by its activator. Early pathologies observed in this experiment were elevated H_2_O_2_ in urine, and higher expression of MCP-1 in the mesangium and podocytes. Interestingly, the activation of AMPK caused an increase of adiponectin excretion from adipocytes [72,73]. A cross-sectional study in a Japanese population showed an inverse relationship between adiponectin and low grade albuminuria in subjects with BMI >25 kg/m^2^ [74,75]. Although obesity is a known risk factor of CKD, there is an inverse correlation between BMI and mortality in this population. This obesity paradox has been observed in numerous studies among patients with GFR <30 mL/min/1.73 m^2^. The studies showed an accumulation of negative processes in patients with low BMI that lead to malnutrition–inflammation–atherosclerosis syndrome [76]. PEW and frailty are prevalent complications of ESRD, with bad prognosis and co-morbidity. One of the causes might be altered adiponectin production and function [52]. A cross-sectional study among 1303 pre-dialysis CKD patients identified high plasma adiponectin as an indicator of PEW. It was most closely linked with lower 24 h creatinine excretion, and thus lower muscle mass [77]. Kaynar et al. [78] observed a similar correlation in patients with advanced CKD, including those on hemodialysis and peritoneal dialysis. Interestingly, PEW was also associated with low fetuin-A concentrations, which is considered in many aspects as opposite to adiponectin. It is important to note that fetuin-A, despite its negative effects on insulin sensitivity and lipid metabolism, is a well-known vascular calcification inhibitor [71,79]. Adiponectin and fetuin-A serum concentrations are inversely correlated, and adiponectin might suppress fetuin-A expression [80,81]. On the contrary, Machiba et al. [82] investigated the associations of nutritional status, adiponectin level, and mortality among hemodialysis patients. Although they also found an inverse association between serum adiponectin and adiposity, there was higher mortality in the high adiponectin group, regardless of truncal fat, total fat, or serum albumin levels. This indicates that adiponectin should not be considered as a mere nutritional marker in patients with CKD. 

## 7. Adiponectin, Vascular Calcifications, and Mineral and Bone Disorders

Although CVD is a main cause of mortality in CKD, in the case of kidney failure, it also has different non-classical risk factors, and is most distinctly associated with vascular calcifications [83]. These are part of wider metabolic complications affecting mineral and bone metabolism. This is due to the kidney’s pivotal role in activated vitamin D, parathormone, phosphatonins, calcium, and phosphorus regulation. CKD related mineral and bone disorders also include abnormalities in bone turnover, mineralization, and mass, causing greater fracture risk [84]. Experimental data have suggested a positive effect of adiponectin upon stabilization of atherosclerotic plaques in various mechanisms [85]. Luo et al. [86] demonstrated in an animal model that adiponectin inhibits the osteoblastic differentiation of vascular smooth muscle cells through AdipoR1. Lu et al. [87] observed a similar positive effect of globular adiponectin in rats, mainly through inhibition of vascular smooth muscle cell apoptosis. Later, Lu and colleagues [88] identified that adiponectin inhibits osteogenesis and calcification in vascular smooth muscle cells. It is mediated by Janus kinase 2/signal transducer and activator of transcription 3 (JAK2/STAT3) pathway inhibition. A meta-analysis of prospective studies, enrolling 14,063 patients with CVD, showed a positive relation between plasma adiponectin and all-cause or cardiovascular mortality. The patients in the highest tertile vs. the lowest tertile of serum baseline adiponectin concentration had 43% higher all-cause mortality, and 69% higher cardiovascular mortality [89]. Aubert et al. [90] performed a cross-sectional study of 197 patients with T2D and calcifying peripheral arteriopathy. Patients with eGFR <30 mL/min/1.73 m^2^ were excluded from the study. The authors found a positive correlation between serum adiponectin and peripheral artery calcification. The authors also analyzed arterial section samples obtained from five patients with ESRD. They found prominent levels of adiponectin in the arterial wall upon immunohistochemical staining. The strongest adiponectin expression was found in the early stages of calcification in medial smooth muscle cells, which might suggest its protective action. Marino et al. [91] analyzed the possible association between the virtual histology intravascular ultrasound (VH-IVUS) features of coronary plaques and serum adiponectin levels in patients with acute coronary syndrome (ACS), or stable angina pectoris (SAP), undergoing coronarography. No correlation was found in the whole group, although serum adiponectin was related to the presence of thin cap fibroatheroma lesions in SAP patients alone. Nomura-Nakayama et al. [92] discovered that HMW adiponectin was inversely correlated with vascular calcification in renal graft recipients. On the other hand, the data from the Korean cohort study for the outcome in patients with chronic kidney disease (KNOW-CKD) study, a large cross-sectional study of pre-dialysis CKD patients, showed that fibroblast growth factor 23 (FGF-23) was associated with coronary artery calcification only in the high serum adiponectin group [93]. Further analysis of this cohort has shown that serum adiponectin in pre-dialysis CKD patients was related with both aortic and arterial stiffness. It was also related with intact parathyroid hormone (iPTH) and alkaline phosphatase (ALP), and inversely related with vitamin D [94]. Sakura et al. [95] analyzed the association between serum adiponectin and vascular calcification in hemodialysis patients without residual kidney function. They found that serum adiponectin was independently related to abdominal aortic calcification. There is growing evidence of the prominent role of adiponectin in bone metabolism and the calcium-phosphate axis. It is assumed that adiponectin causes bone loss in all stages of CKD through various mechanisms. Okuno et al. [96] showed an inversed, independent correlation between bone mass density and serum adiponectin in male hemodialysis patients. There was a relation between serum adiponectin and serum cross-linked *N*-telopeptide of type I collagen, a marker of bone resorption. Rutkowski et al. [97] showed that mice with adiponectin overexpression had higher serum calcium, increased calcinuria, and normal serum and urine phosphate. In this study, adiponectin directly reduced renal Klotho expression and serum FGF-23 levels. There was also a different reaction of the calcium–phosphate axis, after a dietary phosphate or calcium challenge, depending on adiponectin levels. Adiponectin overexpressing mice showed augmented FGF-23 secretion after a dietary phosphate challenge, and higher serum calcium after an increase in dietary calcium. All this showed the direct role of adiponectin in mineral balance.

## 8. Adiponectin as a Marker of Kidney Disease

Adiponectin might be considered as a marker of kidney injury and risk of disease progression. Kollerits et al. [98] showed that high serum adiponectin might be predictive for CKD progression in men, but not in women. A similar correlation between CKD progression and baseline serum adiponectin has been found in patients with type 1 diabetes (T1D) [99]. On the contrary, serum adiponectin and its HMW isoform were not associated with eGFR decline in a prospective cohort study among elderly Japanese without CKD. Interestingly, serum adiponectin was found to be inversely related to hand grip and knee extension strength in this population [100]. Novel trials have assessed the utility of adiponectin as a urine marker of kidney injury. Patients with proteinuria >150 mg/dL excreted significantly larger amounts of the LMW and HMW forms in their urine. Adiponectin in these forms can activate AdipoR1 in proximal tubular cells, imposing an anti-inflammatory effect [35]. One study showed that urinary adiponectin and osteopontin concentrations might be a predictor of kidney injury in children with systemic lupus erythematosus. The authors proved that higher concentrations of these markers correlated with worse biopsy NIH-CI (chronicity index) score and observed eGFR decline. However, the observations were limited to 1 year, and higher levels of these biomarkers predicted chronic disease with moderate accuracy (AUC = 0.75) [101]. Other authors developed an ultrasensitive enzyme-linked immunosorbent assay capable of detecting exceptionally low urinary adiponectin concentrations. The results showed a significantly higher urine adiponectin concentration in patients with diabetes. Furthermore, there was a positive correlation between urinary adiponectin concentration and CKD progression. Moreover, western blot testing showed that, contrary to healthy controls, diabetic patients excreted middle molecular weight (MMW), or even HMW, adiponectin in their urine. The authors suggested that testing the adiponectin concentration in urine, particularly the MMW form, might be more reliable than albumin-to-creatinine ratio (ACR) evaluation [102]. There have been a few trials assessing urinary adiponectin in non-diabetic patients. One of them showed a positive correlation between albuminuria and urinary adiponectin in hypertensive patients treated with olmesartan, which is a blocker of angiotensin II receptor AT1. Studies have also shown that urinary adiponectin levels might predict treatment response. Patients with higher urine adiponectin levels achieved reductions in albuminuria after 16 weeks of diet treatment less often than those with lower adiponectin levels [103]. A similar increase in adiponectin levels in urine and serum has been observed in patients with nephrotic syndrome caused by FSGS. In this clinical situation, urine and serum adiponectins were independently correlated with proteinuria, hypoalbuminaemia, and hyperlipidaemia, although there was no correlation between adiponectin levels and clinical response [104].

## 9. Summary

**Figure 1:**
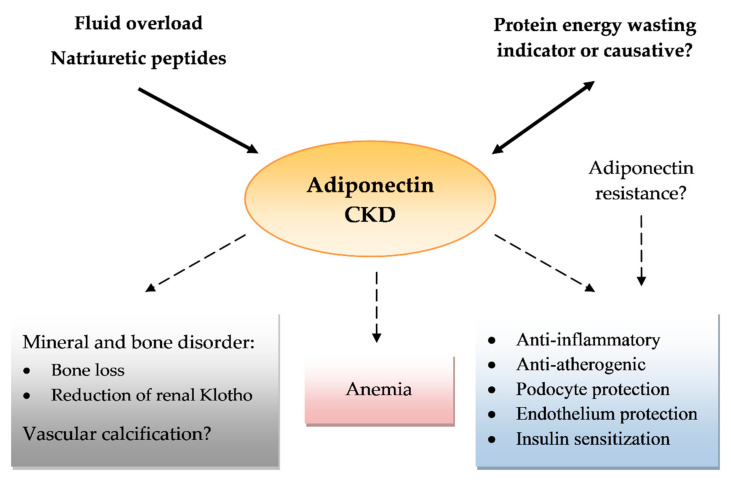
Effects of adiponectin in chronic kidney disease (CKD). Arrows pointing at adiponectin show a role of protein energy wasting, fluid overload, and natriuretic peptides in increasing serum adiponectin levels as a possible confounding factor. A number of question marks reflect the uncertain effects or conflicting results of reports.

The last few years have brought a rising body of evidence that adiponectin is a multipotential protein, with anti-inflammatory, metabolic, anti-atherogenic, and ROS protective actions. These results, which have been acquired from numerous studies in predominantly animal or cell lines, have raised hopes for new therapies for medical conditions such as diabetes, atherosclerosis, or obesity. Similarly, adiponectin has shown many positive and direct actions in kidney diseases and among many kidney cells. In contrast, data from large cross-sectional and cohort studies showed a positive correlation between serum adiponectin and mortality in CKD. The nature of this adiponectin paradox is still unclear. Some authors suggest that increases in adiponectin might strictly be associated with lower renal clearance or might be a compensating mechanism related to the growing number of insults related to kidney function loss. On the other hand, some well-constructed studies have shown an independent correlation between adiponectin and mortality. Adiponectin is also strictly related to PEW, natriuretic peptides, and, in some trials, vascular calcifications, which are all distinct mortality risk factors for CKD. The causal relationship of adiponectin and these complications is still undetermined (Figure 1).

Adiponectin might be considered as a marker of many negative factors in CKD, but novel research has shown an important role of adiponectin in mineral and bone metabolism and vascular calcifications. Adiponectin also plays an important role in metabolism, and its role in malnutrition is uncertain. The most important research on the role of adiponectin in CKD is shown in Table 1. Studies regarding many of these aspects have shown conflicting results, and the possibility of adiponectin receptor agonist treatment [1] needs further elucidation.

The multidirectional involvement of adiponectin in the pathogenesis of kidney diseases is associated with a broad spectrum of challenges. Identifying mechanisms and pathways by which adiponectin is involved in the pathogenesis of kidney diseases is of particular interest and needs further studies. The search for biomarkers that predispose patients to a development of kidney diseases, and that may be helpful in monitoring of clinical course and their progression, will be the focus of research in the coming years. These biomarkers could include new molecules regulating T and B cell activation, proinflammatory chemokines, cytokines, and growth factors, as well as other proteins involved in immune response and fibrosis. The challenge is to develop appropriate strategies to investigate the molecular mechanisms involved in the pathogenesis of kidney diseases. The development of a certain algorithm including the molecular biomarkers could be helpful in the appropriate diagnosis and monitoring of kidney disease progression. Nevertheless, the search for these biomarkers will require further research.

## Figures and Tables

**Table 1:** Selected studies of adiponectin’s role in chronic kidney disease.

Authors and Year of Publication	Study Population	Type of Study	Result of Increased Adiponectin	Reference
Menon et al. 2006	820 CKD stage 3 and 4	Cohort	↑all-cause mortality ↑CV mortality	[59]
Rao et al. 2008	182 HD	Cohort	U shaped relation with all-cause mortality and CVD	[60]
Rhee et al. 2015	501 HD	Cohort	↑all-cause mortality	[61]
Abdallah et al. 2012	133 HD	Cohort	↓all-cause mortality ↓CVD episodes	[62]
Moreno et al. 2016	1426 T2D	Cohort	No significant, independent association with mortality in patients with GFR <60 mL/min/1.73 m^2^	[63]
Kim et al. 2018	2113 CKD stage 1–5, predialysis	Cohort	Anemia	[69]
Hyun et al. 2017	1303 CKD, predialysis	Cross-sectional	Indicator of PEW and ↓muscle mass	[77]
Kaynar et al. 2014	150 Predialysis CKD, HD, PD, KTx, control group	Cross-sectional	Indicator of PEW	[78]
Machiba et al. 2018	113 HD	Cohort	↓adiposity ↑all-cause mortality independent of adiposity	[82]
Hyun et al. 2019	1435 CKD stage 1–5, predialysis	Cross-sectional	↑coronary calcification in high FGF23 group	[93]
Kim et al. 2017	716 CKD stage 1–5, predialysis	Cohort	↑aortic stiffness	[94]
Nomura-Nakayama et al. 2018	51 KTx	Cohort	↓vascular calcification	[92]
Sakura et al. 2017	101 Male HD	Cross-sectional	↑abdominal aortic calcification	[95]
Okuno et al. 2012	114 Male HD	Cross-sectional	↓bone mineral density	[96]
Kollerits et al. 2007	177	Cohort	CKD progression in males	[98]

CKD, chronic kidney disease; CV, cardiovascular; CVD, cardiovascular disease; FGF 23, fibroblast growth factor 23; HD, hemodialysis; KTx, kidney transplant; PD, peritoneal dialysis; PEW, protein–energy wasting; T2D, type 2 diabetes.

## Abbreviations

ACEI	Angiotensin-converting-enzyme inhibitors
ACR	Albumin-to-creatinine ratio
ACS	Acute coronary syndrome
AdipoR1	Adiponectin receptor 1
AdipoR2	Adiponectin receptor 2
*AdipoQ*	Adiponectin gene
AMPK	5′-AMP-activated protein kinase
ALP	Alkaline phosphatase
APPL1	Adaptor protein, phosphotyrosine interacting with PH domain and leucine zipper 1
ARBs	Angiotensin II receptor blockers
BMI	Body mass index
CCL5	C-C motif chemokine ligand 5
CKD	Chronic kidney disease
CVD	Cardiovascular disease
eGFR	Estimated glomerular filtration rate
eNOS	Endothelial nitric oxide synthase
Epac	Exchange protein activated by cAMP
FSGS	Focal segmental glomerulosclerosis
ESRD	End-stage renal disease
FGF-23	Fibroblast growth factor 23
FOXO1	Forkhead box O1
GCSF	Granulocyte colony-stimulating factor
GM-CSF	Granulocyte-macrophage colony-stimulating factor
HD	Hemodialysis
HDL	High-density lipoprotein
HMW	High molecular weight
IL-1R4	Interleukin 1 receptor 4
IL-10	Interleukin 10
IL-12	Interleukin 12
IL-17B	Interleukin 17B
IL-21	Interleukin 21
iPTH	Intact parathyroid hormone
JAK2	Janus kinase 2
KNOW-CKD	KoreaN cohort study for Outcome in patients With Chronic Kidney Disease
LDL	Low-density lipoprotein
LMW	Low molecular weight
MCP-1	Monocyte chemoattractant protein-1
MDRD	Modification of Diet in Renal Disease
MMW	Middle molecular weight
mTOR	Mechanistic target of rapamycin kinase
NF-κB	Nuclear factor κB
NLRP3	NLR family pyrin domain containing 3
NOX	NADPH oxidase
NT-proBNP	*N*-terminal prohormone of brain natriuretic peptide
PEW	Protein-energy wasting
PGC-1α	Peroxisome proliferator-activated receptor γ coactivator-1α
PKA	Protein kinase A
PPAR-α	Peroxisome proliferator-activated receptor α
PPAR-γ	Peroxisome proliferator-activated receptor γ
ROS	Reactive oxygen species
S1P	Sphingosine 1-phosphate
S6K	Ribosomal protein S6 kinase
SAP	Stable angina pectoris
STAT3	Signal transducer and activator of transcription 3
SREBP-1c	Sterol regulatory element binding protein 1c
T1D	Type 1 diabetes
T2D	Type 2 diabetes
TGFβ	Transforming growth factor β
TNF-α	Tumor necrosis factor α
TPO	Thrombopoietin
VEGFR-1	Vascular endothelial growth factor receptor-1
VH-IVUS	Virtual histology intravascular ultrasound
